# Controlling false discoveries in high-dimensional situations: boosting with stability selection

**DOI:** 10.1186/s12859-015-0575-3

**Published:** 2015-05-06

**Authors:** Benjamin Hofner, Luigi Boccuto, Markus Göker

**Affiliations:** 1 Department of Medical Informatics, Biometry and Epidemiology, Friedrich-Alexander-University Erlangen-Nuremberg, Waldstraße 6, Erlangen, 91054 Germany; 2 Greenwood Genetic Center, 113 Gregor Mendel Circle, Greenwood, 29646 SC USA; 3 Leibniz Institute DSMZ – German Collection of Microorganisms and Cell Cultures, Inhoffenstraße 7b, Braunschweig, 38124 Germany

**Keywords:** Boosting, Error control, Variable selection, Stability selection

## Abstract

**Background:**

Modern biotechnologies often result in high-dimensional data sets with many more variables than observations (*n*≪*p*). These data sets pose new challenges to statistical analysis: Variable selection becomes one of the most important tasks in this setting. Similar challenges arise if in modern data sets from observational studies, e.g., in ecology, where flexible, non-linear models are fitted to high-dimensional data. We assess the recently proposed flexible framework for variable selection called stability selection. By the use of resampling procedures, stability selection adds a finite sample error control to high-dimensional variable selection procedures such as Lasso or boosting. We consider the combination of boosting and stability selection and present results from a detailed simulation study that provide insights into the usefulness of this combination. The interpretation of the used error bounds is elaborated and insights for practical data analysis are given.

**Results:**

Stability selection with boosting was able to detect influential predictors in high-dimensional settings while controlling the given error bound in various simulation scenarios. The dependence on various parameters such as the sample size, the number of truly influential variables or tuning parameters of the algorithm was investigated. The results were applied to investigate phenotype measurements in patients with autism spectrum disorders using a log-linear interaction model which was fitted by boosting. Stability selection identified five differentially expressed amino acid pathways.

**Conclusion:**

Stability selection is implemented in the freely available R package stabs (http://CRAN.R-project.org/package=stabs). It proved to work well in high-dimensional settings with more predictors than observations for both, linear and additive models. The original version of stability selection, which controls the per-family error rate, is quite conservative, though, this is much less the case for its improvement, complementary pairs stability selection. Nevertheless, care should be taken to appropriately specify the error bound.

**Electronic supplementary material:**

The online version of this article (doi:10.1186/s12859-015-0575-3) contains supplementary material, which is available to authorized users.

## Background

Variable selection is a notorious problem in many applications. The researcher collects many variables on each study subject and then wants to identify the variables that have an influence on the outcome variable. This problem becomes especially pronounced with modern high-throughput experiments where the number of variables *p* is often much larger than the number of observations *n* (e.g., genomics, transcriptomics, proteomics, metabolomics, metabonomics and phenomics; see, [1-6]) or in complex modeling situations with many potential predictors, where the aim is to find a meaningful non-linear model (see e.g., [7]). One of the major aims in the analysis of these high-dimensional data sets is to detect the signal variables *S*, while controlling the number of selected noise variables *N*. Stepwise regression models are a standard approach to variable selection in settings with relatively few variables. However, even in this case this approach is known to be very unstable (see e.g., [8-10]). Recent approaches that try to overcome this problem and can also be used in high-dimensional settings with *n*≪*p* include penalized regression approaches such as the lasso [11,12], elastic net [13], and boosting [14], or tree based approaches such as random forests [15,16]. More recently, Meinshausen and Bühlmann [17] proposed stability selection, an approach based on resampling of the data set which can be combined with many selection procedures and is especially useful in high-dimensional settings. Shah and Samworth [18] extended the framework by using complementary pairs subsampling and derived less conservative error bounds (“complementary pairs stability selection”). Stability selection has since been widely used, e.g. for gene regulatory network analysis [19,20], in genome-wide association studies [21], graphical models [22,23] or even in ecology [24]. In most publications, stability selection is used in combination with lasso or similar penalization approaches. Here, we discuss the combination of stability selection with component-wise functional gradient descent boosting [25]. Boosting can be easily applied to many data situations: It can be applied to Gaussian regression models, models for count data or survival data, and equally easy to quantile or expectile regression models (for an overview see, [26,27]). Furthermore, it allows one to specify competing effects, which are subject to selection, more freely and flexibly. One can specify simple linear effects, penalized effects for categorical data [28], smooth effects [29], cyclic or monotonic effects [30,31] or spatial effects [7] to name just a few. All these effect types can be freely combined with any type of model. For details on functional gradient descent boosting, see [26,27].

We will provide a short, rather non-technical introduction to boosting in the next section. Stability selection, which controls the per-family error rate, will be introduced, and we also give an overview on common error rates and some guidance on the choice of the parameters in stability selection. An empirical evaluation of boosting with stability selection is presented. In our case study we will examine autism spectrum disorder (ASD) patients and compare them to healthy controls using the boosting approach in conjunction with stability selection. The aim is to detect differentially expressed phenotype measurements. More specifically, we try to assess which amino acid pathways differ between healthy subjects and ASD patients.

## Methods

### A short introduction to boosting

Consider a generalized linear model

(1) $$ {\mathbb{E}}(y|\mathbf{x}) = h(\eta(\mathbf{x}))  $$

with outcome *y*, appropriate response function *h* and linear predictor *η*(*x*). Let the latter be defined as

(2) $$ \eta(\mathbf{x}) = \beta_{0} + \sum_{j = 1}^{p} \beta_{j} x_{j},  $$

with covariates **x**=(*x*_1_,…,*x*_*p*_), and corresponding effects *β*_*j*_, *j*=0,…,*p*. Model fitting aims at minimizing the expected loss $\mathbb {E}(\rho (y, \eta (\mathbf {x})))$ with an appropriate loss function *ρ*(*y*,*η*(**x**)). The loss function is defined by the fitting problem at hand. Thus, for example, Gaussian regression models, i.e. least squares regression models, aim to minimize the squared loss *ρ*(*y*,*η*(**x**))=(*y*−*η*(**x**))^2^. Generalized linear models can be obtained by maximizing the log-likelihood or, analogously, by minimizing the negative log-likelihood function. Logistic regression models with binary outcome, for example, can be fitted by using the negative binomial log-likelihood

$$\begin{aligned} \rho(y, \eta(\mathbf{x})) =& -y \log(P(y = 1 | \eta(\mathbf{x}))) \\ &+(1 - y) \log (1 - P(y = 1 | \eta(\mathbf{x}))) \end{aligned} $$

as loss function or a reparametrization thereof [26]. Further extensions that are not based on a likelihood, such as quantile or expectile regression models [32,33], models for the robust Huber loss [27,34] or survival models that are fitted by directly optimizing the concordance index [35] can be obtained by the use of an appropriate loss function.

In practice, one cannot minimize the expected loss function. Instead, we optimize the empirical risk function

(3) $$ \mathcal{R}(\mathbf{y}, \mathbf{X}) = n^{-1} \sum_{i=1}^{n} \rho(y_{i}, \eta(\mathbf{x}_{i}))  $$

with observations **y**=(*y*_1_,…,*y*_*n*_)^⊤^ and $\mathbf {X} = \left (\mathbf {x}^{\top }_{1}, \ldots \right.,\left. \mathbf {x}^{\top }_{n}\right)^{\top }$. This can be done for arbitrary loss functions by component-wise functional gradient descent boosting [25]. The algorithm is especially attractive owing to its intrinsic variable selection properties [7,28].

One begins with a constant model $\hat {\eta }^{[0]}(\mathbf {x}_{i}) \equiv 0$ and computes the residuals $\mathbf {u}^{[1]} = (u_{1}^{[1]}, \ldots, u_{n}^{[1]})^{\top }$ defined by the negative gradient of the loss function

(4) $$ u_{i}^{[m]} := - \left. \frac{\partial \rho(y_{i}, \eta)}{\partial \eta} \right|_{\eta = \hat{\eta}^{[m-1]}(\mathbf{x}_{i})}  $$

evaluated at the fit of the previous iteration $\hat {\eta }^{[m-1]}(\mathbf {x}_{i})$ (see, [25,26,36]). Each variable *x*_1_,…,*x*_*p*_ is fitted separately to the residuals **u** ^[*m*]^ by least squares estimation (this is called the “base-learner”), and only the variable *j* ^∗^ that describes these residuals best is updated by adding a small percentage *ν* of the fit $\hat {\beta }_{j^{*}}$ (e.g., *ν*=10*%*) to the current model fit, i.e.,

$$ \hat{\eta}^{[m]} = \hat{\eta}^{[m-1]} + \nu \cdot \hat{\beta}_{j^{*}}. $$

New residuals **u** ^[*m*+1]^ are computed, and the whole procedure is iterated until a fixed number of iterations *m*=*m*_stop_ is reached. The final model $\hat {\eta }^{[m_{\text {stop}}]}(\mathbf {x}_{i})$ is defined as the sum of all models fitted in this process. Instead of using linear base-learners (i.e., linear effects) to fit the negative gradient vector **u** ^[*m*]^ in each boosting step, one can also specify smooth base-learners for the variables *x*_*j*_ (see e.g. [29]), which are then fitted by penalized least squares estimation. This allows to fit generalized additive models GAMs; [37,38]) with non-linear effects or even very complex models such as structured additive regression (STAR) models [31,39] with spatio-temporal effects, models with smooth interaction surfaces, cyclic effects, monotonic effects, and so on. In all these models, each modeling component is specified as a separate base-learner. As we update only one base-learner in each boosting iteration, variables or effect types are selected by stopping the boosting procedure after an appropriate number of iterations (“early stopping”). This number is usually determined using cross-validation techniques (see e.g., [40]).

### Stability selection

A problem of many statistical learning approaches including boosting with early stopping is that despite regularization one often ends up with relatively rich models [17,40]. A lot of noise variables might be erroneously selected. To improve the selection process and to obtain an error control for the number of falsely selected noise variables Meinshausen and Bühlmann [17] proposed stability selection, which was later enhanced by Shah and Samworth [18]. Stability selection is a versatile approach, which can be combined with all high-dimensional variable selection approaches. It is based on sub-sampling and controls the *per-family error rate* $\mathbb {E}(V)$, where *V* is the number of false positive variables (for more details on error rates see Additional file 1, Section A.1).

Consider a data set with *p* predictor variables *x*_*j*_, *j*=1,…,*p* and an outcome variable *y*. Let *S*⊆{1,…,*p*} be the set of signal variables, and let *N*⊆{1,…,*p*}/*S* be the set of noise variables. The set of variables that are selected by the statistical learning procedure is denoted by $\hat {S}_{n} \subseteq \{1, \ldots, p\}$. This set $\hat {S}_{n}$ can be considered to be an estimator of *S*, based on a data set with *n* observations. In short, for stability selection with boosting one proceeds as follows:

1. Select a random subset of size ⌊*n*/2⌋ of the data, where ⌊*x*⌋ denotes the largest integer ≤*x*.
2. Fit a boosting model and continue to increase the number of boosting iterations *m*_stop_ until *q* base-learners are selected. $\hat {S}_{\lfloor n/2 \rfloor,\, b}$ denotes the set of selected variables.
3. Repeat the steps 1) and 2) for *b*=1,…,*B*.
4. Compute the relative selection frequencies
  (5) $$ \hat{\pi}_{j} := \frac{1}{B} \sum_{b = 1}^{B} \mathbb{I}_{\{j \in \hat{S}_{\lfloor n/2 \rfloor,\, b}\}}  $$
  per variable (or actually per base-learner).
5. Select all base-learners that were selected with a frequency of at least *π*_thr_, where *π*_thr_ is a pre-specified threshold value. Thus, we obtain a set of *stable variables* $\hat {S}_{\text {stable}} := \{j: \hat {\pi }_{j} \geq \pi _{\text {thr}}\}$.

Meinshausen and Bühlmann [17] show that this selection procedure controls the per-family error rate (*P* *F* *E* *R*). An upper bound is given by

(6) $$ \mathbb{E}(V) \leq \frac{q^{2}}{(2\pi_{\text{thr}} - 1) p}  $$

where *q* is the number of selected variables per boosting run, *p* is the number of (possible) predictors and *π*_thr_ is the threshold for selection probability. The theory requires two assumptions to ensure that the error bound holds:

(i) The distribution $\left \{\mathbb {I}_{\{j \in \hat {S}_{\text {stable}}\}}, j \in N\right \}$ needs to be exchangeable for all noise variables *N*.
(ii) The original selection procedure, boosting in our case, must not be worse than random guessing.

In practice, assumption (i) essentially means that each noise variable has the same selection probability. Thus, all *noise variables* should, for example, have the same correlation with the signal variables (and the outcome). For examples of situations where exchangeability is given see Meinshausen and Bühlmann [17]. Assumption (ii) means that signal variables should be selected with higher probability than noise variables. This assumption is usually not very restrictive as we would expect it to hold for any sensible selection procedure.

**Complementary pairs stability selection** Shah and Samworth [18] introduced a modification of the original stability selection approach. First, they use complementary pairs, i.e., they split the sample *B* times in random halves and each time use both subsamples. Second, they derive an error bound which does not require assumptions (i) and (ii) to hold. This comes at the price that one can only obtain error control for the *expected number of selected variables with low selection probability*

(7) $$ \mathbb{E}(|\hat{S}_{\text{stable}} \cap L_{\theta}|),  $$

where $\hat {S}_{\text {stable}}$ denotes the set of variables selected by stability selection, and $L_{\theta } = \{j: \hat {\pi }_{j} \leq \theta \}$ denotes the set of variables that have a low selection probability in one boosting run on a subsample of size ⌊*n*/2⌋. (An interpretation and a discussion of this error rate is given in Additional file 1, Section A.2.1).

Finally, Shah and Samworth [18] derive stricter error bounds given some assumptions on the selection probabilities of the base-learners, which usually hold:

1. A worst case error bound without further assumptions that equals the error bound given by Meinshausen and Bühlmann [17].
2. A tighter error bound that assumes that the simultaneous selection probabilities, i.e., the probability that the base-learner is selected in both complementary pairs, have a unimodal probability distribution for all *j*∈*L*_*θ*_.
3. The tightest error bound assumes that the simultaneous selection probabilities have an r-concave probability distribution with $r = -\frac {1}{2}$ and that the selection probabilities $\hat {\pi }_{j}$ have an r-concave probability distribution with $r = -\frac {1}{4}$ for all *j*∈*L*_*θ*_.

For a rigorous definition of the assumptions and the derived error bounds as well as an interpretation see [18] and Additional file 1, Section A.2.

**Choice of parameters** The stability selection procedure mainly depends on two parameters: the number of selected variables per boosting model *q* and the threshold value for stable variables *π*_thr_. Meinshausen and Bühlmann [17] propose to chose *π*_thr_∈(0.6,0.9) and claim that the threshold has little influence on the selection procedure. In general, any value ∈(0.5,1) is potentially acceptable, i.e. a variable should be selected in more than half of the fitted models in order to be considered stable. The number of selected variables *q* should be chosen so high that in theory all signal variables *S* can be chosen. If *q* was too small, one would inevitably select only a small subset of the signal variables *S* in the set $\hat {S}_{\text {stable}}$ as $|\hat {S}_{\text {stable}}| \leq |\hat {S}_{\lfloor n/2 \rfloor,\, b}| = q$ (if *π*_thr_>0.5).

The choice of the number of subsamples *B* is of minor importance as long as it is large enough. Meinshausen and Bühlmann [17] propose to use *B*=100 replicates, which seems to be sufficient for an accurate estimation of $\hat {\pi }_{j}$ in most situations.

In general, we would recommend to choose an upper bound *P* *F* *E* *R*_max_ for the *P* *F* *E* *R* and specify either *q* or *π*_thr_, preferably *q*. The missing parameter can then be computed from Equation (6), where equality is assumed. For a fixed value *q*, we can easily vary the desired error bound *P* *F* *E* *R*_max_ by varying the threshold *π*_thr_ accordingly. As we do not need to re-run the subsampling procedure, this is very easy and fast. In a second step, one should check that the computed value is sensible, i.e. that *π*_thr_∈(0.5,1), or that *q* is not too small, or that *P* *F* *E* *R*_max_ is not too small or too large. Note that the *P* *F* *E* *R* can be greater than one as it resembles the tolerable expected number of falsely selected noise variables. An overview on common error rates is given in Additional file 1 (Section A.1), where we also give some guidance on the choice of *P* *F* *E* *R*_max_.

The size of the subsamples is no tuning parameter but should always be chosen to be ⌊*n*/2⌋. This an essential requirement for the derivation of the error bound (6) as can be seen in the proof of Lemma 2 [17], which is used to prove the error bound. Other (larger) subsample sizes would theoretically be possible but would require the derivation of a different error bound for that situation.

### Simulation study

To evaluate the impact of the tuning parameters *q* and *π*_thr_, the upper bound *P* *F* *E* *R*_max_, and the assumptions for the computation of the upper bound on the selection properties, we conducted a simulation study using boosting in conjunction with stability selection. Additionally, we examined the impact of the characteristics of the data set on the performance. We considered two scenarios: First, we used a logistic regression model with linear effects. Second, we used a Gaussian regression model with non-linear effects, i.e., a generalized additive model (GAM).

**Linear logistic regression model** We considered a classification problem with a binary outcome variable. The data were generated according to a linear logistic regression model with linear predictor *η*=**X** *β* and

$$ Y \sim \text{Binom}\left(\frac{\exp(\eta)}{1 + \exp(\eta)}\right). $$

The observations *x*_*i*_=(*x*_*i*1_,…,*x*_*ip*_), *i*=1,…,*n* were independently drawn from

$$ x \sim \mathcal{N}(0, \Sigma), $$

and gathered in the design matrix **X**. We set the number of predictor variables to *p*∈{100,500,1000}, and the number of observations to *n*∈{50,100,500}. The number of influential variables varied within *p*_infl_∈{2,3,8}, where *β*_*j*_ was sampled from {−1,1} for an influential variable and set to zero for all non-influential variables. We used two settings for the design matrix:

1. independent predictor variables, i.e. *Σ*=**I**,
2. correlated predictor variables drawn from a Toeplitz design with covariance matrix *Σ*_*kl*_=0.9^|*k*−*l*|^, *k*,*l*=1,…,*p*.

For each of the data settings we used all three error bounds in combination with varying parameters *q*∈{4,8,12,16,20}, and *P* *F* *E* *R*_max_∈{0.05,1,2,5}. We used *B*=50 complementary pairs, i.e., 2*B* subsamples in total. Each simulation setting was repeated 50 times.

**Gaussian additive regression model** We considered a regression problem with linear and smooth covariate effects. The data were generated according to a Gaussian additive model with additive predictor $\eta = \sum _{i} f_{i}(x_{i})$ and

$$ Y \sim \mathcal{N}\left(\eta, \sigma^{2}\right), $$

where the variance *σ* ^2^ was chosen for each setting such that explained variation *R* ^2^≈0.33. The observations *x*_*i*_=(*x*_*i*1_,…,*x*_*ip*_), *i*=1,…,*n* were independently drawn from a uniform distribution $x \sim \mathcal {U}(-2, 2)$, and gathered in the design matrix **X**. We used two settings for the design matrix:

1. independent uniform predictor variables,
2. correlated uniform predictor variables drawn from a Toeplitz design with correlation matrix *ρ*_*kl*_=0.9^|*k*−*l*|^, *k*,*l*=1,…,*p*.

**Figure 1:**
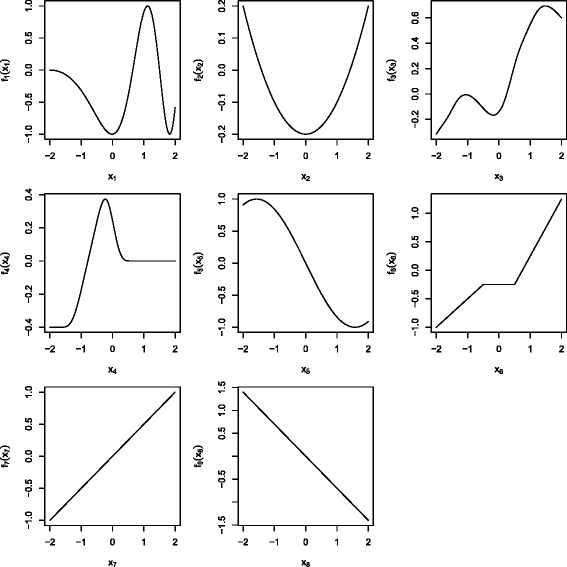
Covariate effects. Effect types range from oscillating functions (*f*_1_), over quadratic functions (*f*_2_), arbitrary smooth function (*f*_3_ and *f*_4_), cosine functions (*f*_5_), and piecewise linear functions (*f*_6_), to linear functions (*f*_7_ and *f*_8_). For two influential covariates we used *f*_1_ and *f*_2_, for three influential covariates we used *f*_1_ to *f*_3_ and for eight influential covariates we used all functions.

We set the number of predictor variables to *p*∈{50,100,200}, and the number of observations to *n*∈{100,500,1000}. The number of influential variables varied within *p*_infl_∈{2,3,8}. The effects of the influential variables are depicted in Figure 1. All other effects were set to zero.

As above, we considered for each of the data settings all three error bounds in combination with varying parameters *q*∈{4,8,12,16,20}, and *P* *F* *E* *R*_max_∈{0.05,1,2,5}. We used *B*=50 complementary pairs, i.e., 2*B* subsamples in total. Each simulation setting was repeated 50 times.

### Case study: differential phenotype expression for ASD patients versus controls

We examined autism spectrum disorder (ASD) patients [41] and compared them to healthy controls. The aim was to detect differentially expressed amino acid pathways, i.e. amino acid pathways that differ between healthy subjects and ASD patients [42]. We used measurements of absorbance readings from Phenotype Microarrays developed by Biolog (Hayward, CA). The arrays are designed so as to expose the cells to a single carbon energy source per well and evaluate the ability of the cells to utilize this energy source to generate NADH [43]. The array plates were incubated for 48 h at 37°C in 5% CO2 with 20,000 lymphoblastoid cells per well. After this first incubation, Biolog Redox Dye Mix MB was added (10 *μ*L/well) and the plates were incubated under the same conditions for an additional 24 h. As the cells metabolize the carbon source, tetrazolium dye in the media is reduced, producing a purple color according to the amount of NADH generated. At the end of the 24 h incubation, the plates were analyzed utilizing a microplate reader with readings at 590 and 750 nm. The first value (A_590_) indicated the highest absorbance peak of the redox dye and the second value (A_750_) gave a measure of the background noise. The relative absorbance (A _590−750_) was calculated per well.

Each row of the data set described the measurement of *one well per biological replicate*. With *n*=35 biological replicates (17 ASD patients and 18 controls) and *p*=4·96=384 wells we thus theoretically got *n*·*p*=13440 observations. Due to one missing value the data set finally contained only 13439 observations. The data is available as a supplement to Boccuto *et al.* [42] and in the R package opm [44-46], which was also used to store, manage and annotate the data set.

For all available biological replicates we obtained the amino acid annotation for each measurement in that replicate, i.e. we set up an incidence vector per observation for all available peptides. The incidence vector was one if the peptide contained that amino acid and zero if it did not. We ended up with 27 amino acid occurrence annotations in total (including some non-proteinogenic amino acids). In the next step, we modeled the differences of the measured values between ASD patients and controls to assess which amino acid pathways were differentially expressed. Therefore we set up a model of the following form:

$$\begin{array}{*{20}l} {}\log(y) = \beta_{0} + \beta_{1} \text{group} & + b_{\text{id}} + \beta_{2,1} I_{\text{P1}} + \beta_{2,2} I_{\text{P2}} + \ldots + \\ & + X(\text{group}) \cdot \widetilde{b}_{\text{id}} \,+ \\ & + X(\text{group}) \cdot \beta_{3,1} I_{\text{P1}} + \\& + X(\text{group}) \cdot \beta_{3,2} I_{\text{P2}} + \ldots, \end{array} $$

where *y* was the measured PM value, *β*_0_ was an overall intercept, *β*_1_ was the overall group effect (the difference between ASD patients and controls irrespective of the amino acid that the measurement belonged to). Additionally, we used an random effect for the replicate (*b*_ID_) to account for subject-specific effects. The amino acid effects *β*_2,*j*_ represent the differences of the log(*y*) values between amino acid, as *I*_P*j*_ is an indicator function, which was 0 if the well did not belong to amino acid *j*, and 1 if it did; this means we obtained dummy-coded effect estimates from the first line of the model formula.

The most interesting part was given by the second and third line of the model: *X*(group) was a group-specific function which was either −1 for controls or 1 for ASD cases. We used this sum-to-zero constraint in an interaction with dummy-coded amino acid effects. The coefficients *β*_3,*j*_ hence represented the deviation of the groups from the global effect of the *j*th amino acid. If *β*_3,*j*_=0, no group-specific effect was present, i.e. the amino acid did not differ between the groups. If *β*_3,*j*_≠0, the difference between the two groups was twice this effect, i.e. *X*(ASD)·*β*_3,*j*_−(*X*(Control)·*β*_3,*j*_)=1·*β*_3,*j*_−(−1·*β*_3,*j*_)=2*β*_3,*j*_. Note that we also specified a group-specific random effect $\widetilde {b}_{\text {ID}}$.

First, we fitted an offset model containing all main effects, i.e. we modeled differences in the maximum curve height with respect to different amino acids while neglecting possible differences in amino acid effects between groups. In a second step, we started from this offset model and additionally allowed for interactions between the group and the amino acids, while keeping the main effects in the list of possible base-learners, and checked if any interactions were present. These represent differential PM expressions between groups.

In total, we ended up with 57 base-learners (group effect, main amino acid effects, group-specific effects, and an overall and a group-specific random effect). All models were fitted using boosting. The selection of differentially expressed amino acids was done using stability selection. We set the number of selected variables per boosting model to *q*=10 and chose an upper bound for the *P* *F* *E* *R*≤1. To judge the magnitude of the multiplicity correction, we related the used *P* *F* *E* *R* to the significance level *α*, i.e. the standard *P* *C* *E* *R*: The upper bound for the *P* *F* *E* *R* equaled *α*=1/57=0.0175 in this setting. With the unimodality assumption, this led to a cutoff *π*_thr_=0.87. With the r-concavity assumption, the error bound was *π*_thr_=0.69, while the error bound became *π*_thr_=1 without assumptions. Subsequently we used cross-validation to obtain the optimal stopping iteration for the model. The code for model fitting and stability selection is given as an electronic supplement [see Additional file 2].

## Results and discussion

### Simulation study

**Figure 2:**
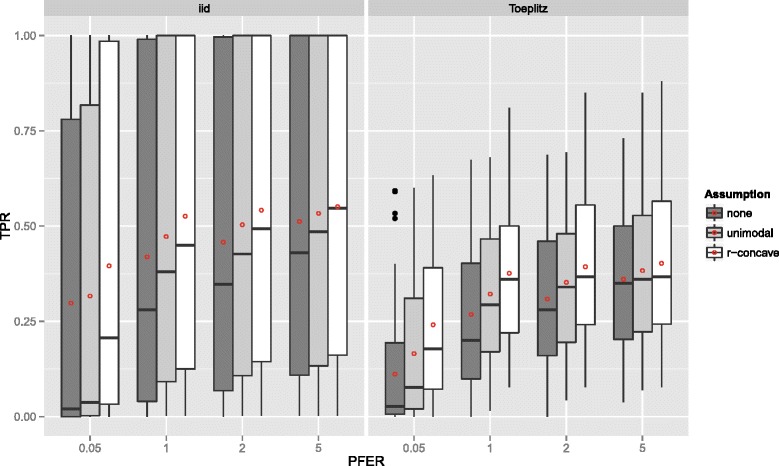
True positives rates – Linear logistic regression model. Boxplots for the true positives rates (TPR) for all simulation settings with separate boxplots for the correlation settings (independent predictor variables or Toeplitz design), *P* *F* *E* *R*_max_ and the assumption used to compute the error bound. Each observation in the boxplot is the average of the 50 simulation replicates. The open red circles represent the average true positive rates.

**Linear logistic regression model** Figure 2 displays the true positive rates for different *P* *F* *E* *R*_max_ bounds, the three assumptions (E1) to (E3) and for the two correlation schemes. Different sizes of the data set (*n* and *p*) as well as different numbers of true positives (*p*_infl_) were not depicted as separate boxplots. For each upper bound *P* *F* *E* *R*_max_ and each data situation (uncorrelated/Toeplitz), the true positive rate (TPR) increased with stronger assumptions (E1) to (E3). The true positive rate was lower when the predictors were correlated.

**Figure 3:**
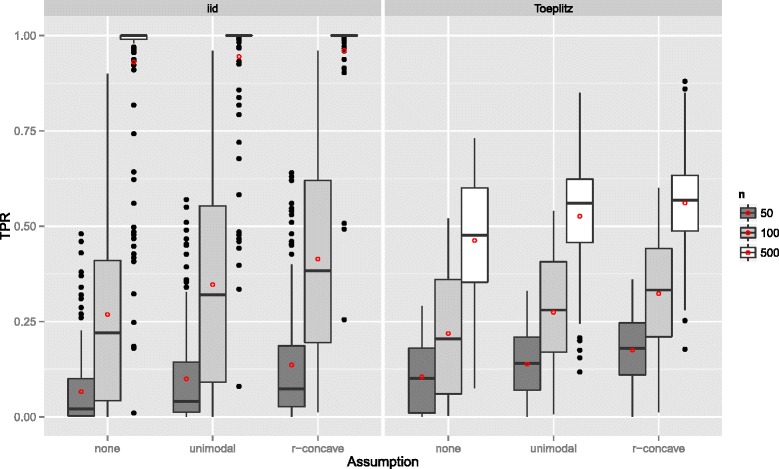
True positives rates by the number of observations *n* – Linear logistic regression model. Boxplots for the true positives rates (TPR) for all simulation settings with separate boxplots for different numbers of observations (*n*), the correlation settings (independent predictor variables or Toeplitz design), and the assumptions used to compute the error bound. Each observation in the boxplot is the average of the 50 simulation replicates. The open red circles represent the average true positive rates.

**Figure 4:**
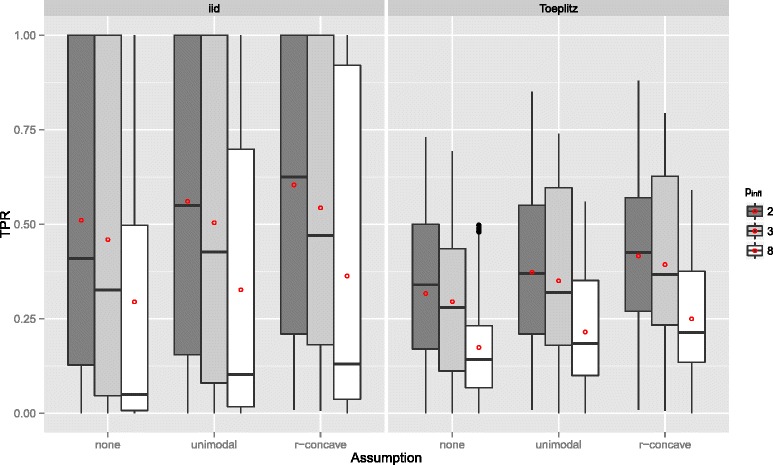
True positives rates by the number of influential variables *p*_infl_ – Linear logistic regression model. Boxplots for the true positives rates (TPR) for all simulation settings with separate boxplots for different numbers of influential variables (*p*_infl_), the correlation settings (independent predictor variables or Toeplitz design), and the assumptions used to compute the error bound. Each observation in the boxplot is the average of the 50 simulation replicates. The open red circles represent the average true positive rates.

**Figure 5:**
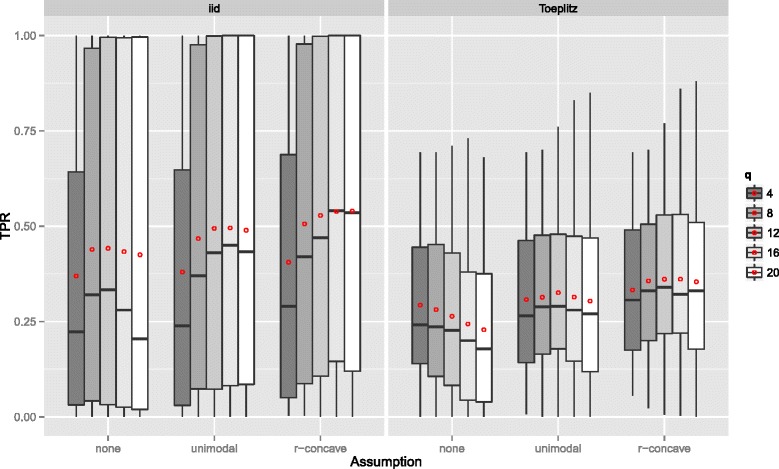
True positives rates by the number of selected variables per boosting run *q* – Linear logistic regression model. Boxplots for the true positives rates (TPR) for all simulation settings with separate boxplots for different numbers of selected variables per boosting run (*q*), the correlation settings (independent predictor variables or Toeplitz design), and the assumptions used to compute the error bound. Each observation in the boxplot is the average of the 50 simulation replicates. The open red circles represent the average true positive rates.

If the number of observations *n* increased, the TPR increased as well with more extreme cases for uncorrelated predictors (Figure 3). With very few observations (*n*=50), the TPR was generally very small. Considering the size of the subsamples, which is equal to 25, this is quite natural. Recently, [47] advocated to increase the sample size of the subsamples from ⌊*n*/2⌋ to larger values to avoid biased selection of base-learners due to too small samples. Yet, as discussed above, this is currently not possible, as one would need to derive a different error bound for that situation. Conversely, the TPR decreases with an increasing number of truly influential variables *p*_infl_ (Figure 4). The number of selected variables per boosting run *q* is less important (Figure 5), as long as it is large enough to result in enough variables *q* to be selected and not too large so that too many variables would be selected in each run.

**Figure 6:**
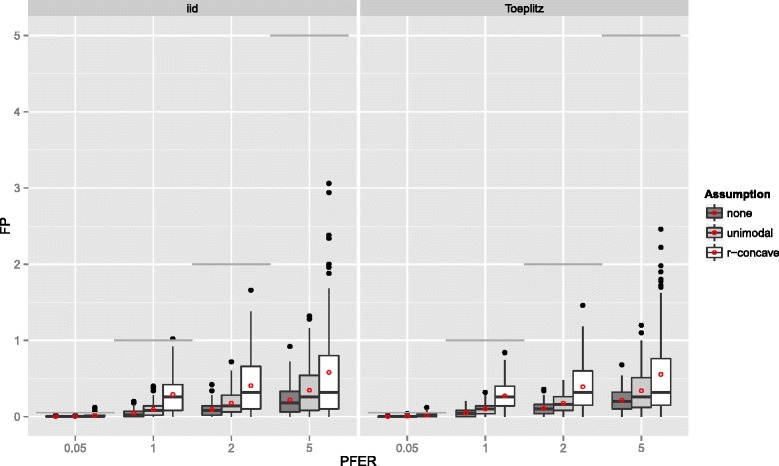
Number of false positives – Linear logistic regression model. Boxplots for the number of false positives (FP) for all simulation settings with separate boxplots for the correlation settings (independent predictor variables or Toeplitz design), *P* *F* *E* *R*_max_ and the assumption used to compute the error bound. Each observation in the boxplot is the average of the 50 simulation replicates. The open red circles represent the average number of false positives. The gray horizontal lines represent the error bounds.

The number of false positives, which is bounded by the upper bound for the per-family error rate, is depicted in Figure 6. Overall, the error rate seemed to be well controlled with very few violations of the less conservative bounds in the settings with an error bound of 0.05 and r-concavity assumption. Especially the standard error bound (E1) seemed to be conservatively controlled. The average number of false positives increased with increasing *P* *F* *E* *R*_max_ and with stronger distributional assumptions on the simultaneous selection probabilities. In general, one should note that stability selection is quite conservative as it controls the *P* *F* *E* *R*. The given upper bounds for the *P* *F* *E* *R* corresponded to per-comparison error rates between 0.05 and 0.00005.

**Figure 7:**
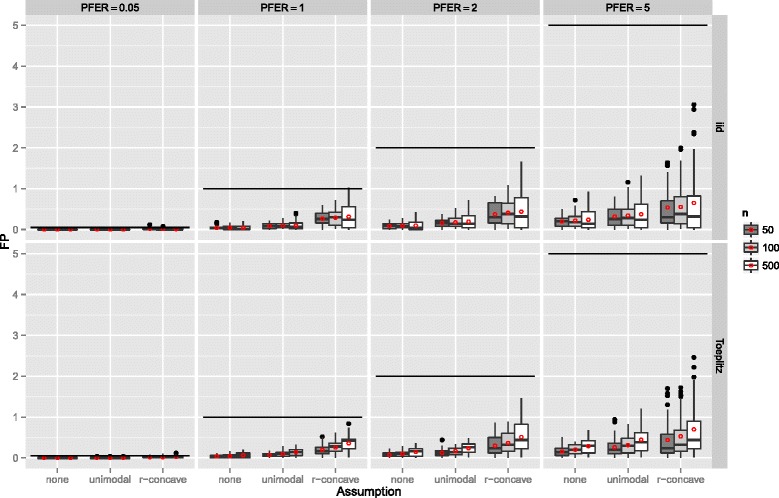
Number of false positives by the number of observations *n* – Linear logistic regression model. Boxplots for the number of false positives (FP) for all simulation settings with separate boxplots for different numbers of observations (*n*), the correlation settings (independent predictor variables or Toeplitz design), the *P* *F* *E* *R*, and the assumptions used to compute the error bound. Each observation in the boxplot is the average of the 50 simulation replicates. The open red circles represent the average number of false positives.

**Figure 8:**
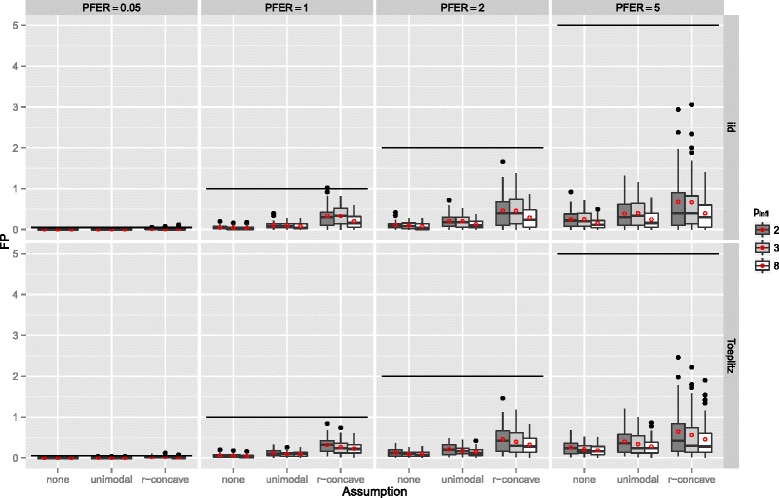
Number of false positives by the number of influential variables *p*_infl_ – Linear logistic regression model. Boxplots for the number of false positives (FP) for all simulation settings with separate boxplots for different numbers of influential variables (*p*_infl_), the correlation settings (independent predictor variables or Toeplitz design), the *P* *F* *E* *R*, and the assumptions used to compute the error bound. Each observation in the boxplot is the average of the 50 simulation replicates. The open red circles represent the average number of false positives.

**Figure 9:**
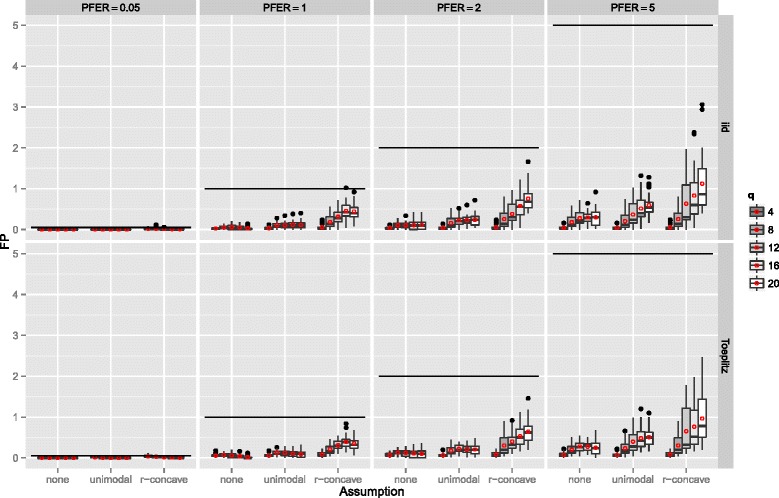
Number of false positives by the number of selected variables per boosting run *q* – Linear logistic regression model. Boxplots for the number of false positives (FP) for all simulation settings with separate boxplots for different numbers of selected variables per boosting run (*q*), the correlation settings (independent predictor variables or Toeplitz design), the *P* *F* *E* *R*, and the assumptions used to compute the error bound. Each observation in the boxplot is the average of the 50 simulation replicates. The open red circles represent the average number of false positives.

If the number of observations *n* increased, the number of false positives stayed constant or increased slightly and the variability increased as well (Figure 7). The number of false positives showed a tendency to decrease with an increasing number of truly influential variables *p*_infl_ (Figure 8). If the number of selected variables per boosting run *q* was small, i.e., only highly frequently selected variables were considered to be stable, the number of false positives decreased (Figure 9). This observation is somehow contrary to the optimal choices of *q* with respect to the true positive rate. However, an optimal true positive rate is more important than a low number of false positives as long as the error rate is controlled.

**Figure 10:**
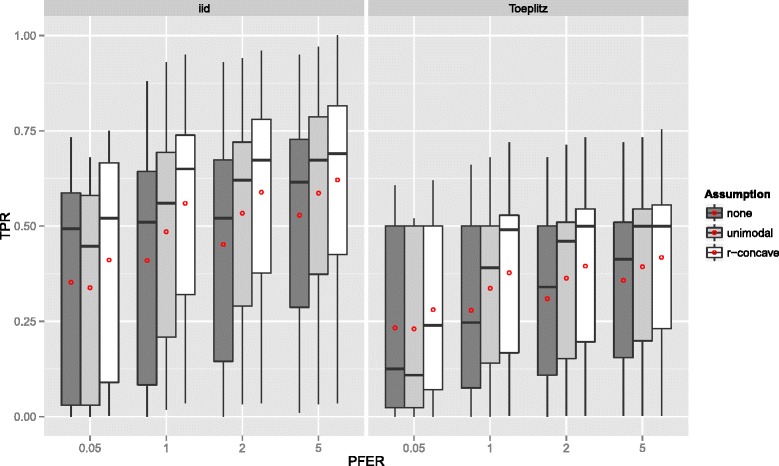
True positives rates – Gaussian additive regression model. Boxplots for the true positives rates (TPR) for all simulation settings with separate boxplots for the correlation settings (independent predictor variables or Toeplitz design), *P* *F* *E* *R*_max_ and the assumption used to compute the error bound. Each observation in the boxplot is the average of the 50 simulation replicates. The open red circles represent the average true positive rates.

**Figure 11:**
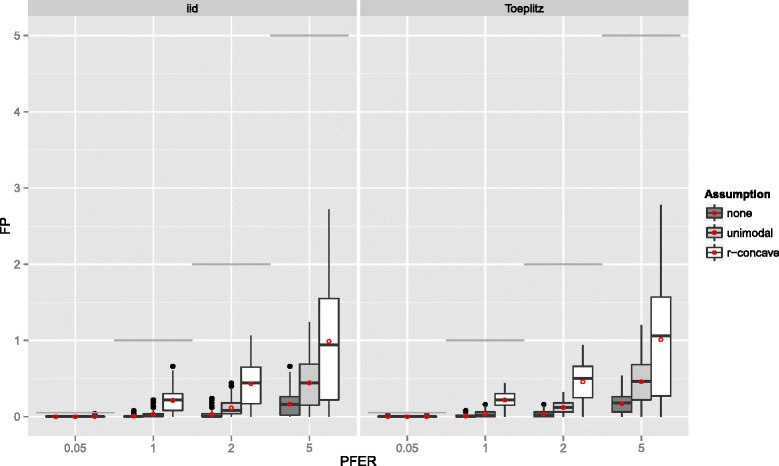
Number of false positives – Gaussian additive regression model. Boxplots for the number of false positives (FP) for all simulation settings with separate boxplots for the correlation settings (independent predictor variables or Toeplitz design), *P* *F* *E* *R*_max_ and the assumption used to compute the error bound. Each observation in the boxplot is the average of the 50 simulation replicates. The open red circles represent the average number of false positives. The gray horizontal lines represent the error bounds.

**Figure 12:**
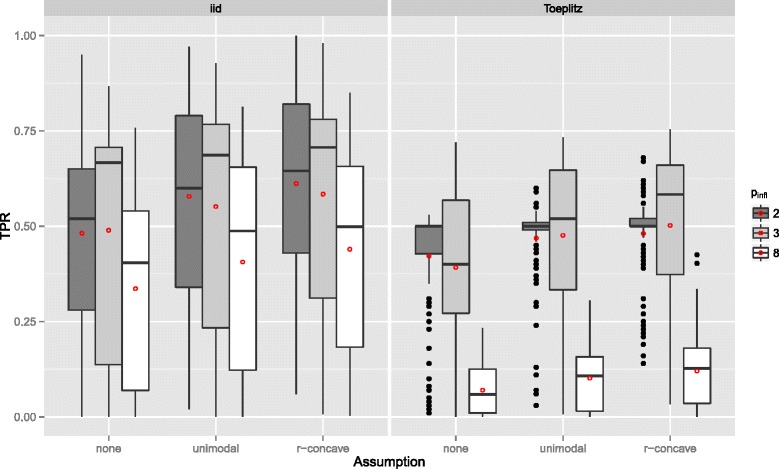
True positives rates by the number of influential variables *p*_infl_ – Gaussian additive regression model. Boxplots for the true positives rates (TPR) for all simulation settings with separate boxplots for different numbers of influential variables (*p*_infl_), the correlation settings (independent predictor variables or Toeplitz design), and the assumptions used to compute the error bound. Each observation in the boxplot is the average of the 50 simulation replicates. The open red circles represent the average true positive rates.

**Figure 13:**
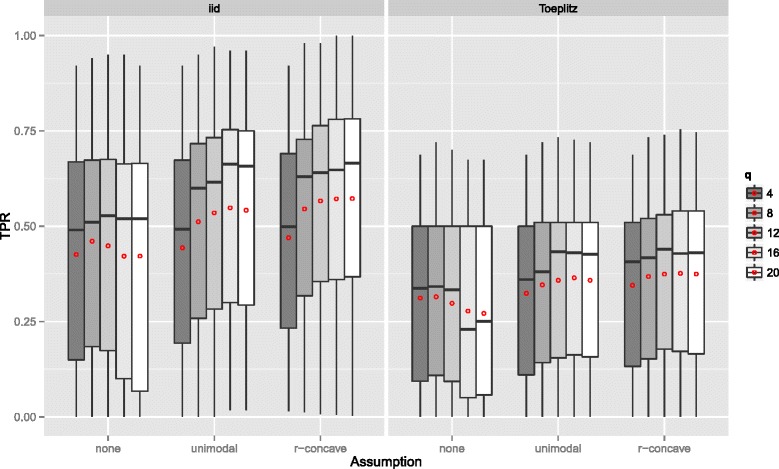
True positives rates by the number of selected variables per boosting run *q* – Gaussian additive regression model. Boxplots for the true positives rates (TPR) for all simulation settings with separate boxplots for different numbers of selected variables per boosting run (*q*), the correlation settings (independent predictor variables or Toeplitz design), and the assumptions used to compute the error bound. Each observation in the boxplot is the average of the 50 simulation replicates. The open red circles represent the average true positive rates.

**Gaussian additive regression model** The results of the Gaussian additive model are essentially the same. Yet, both the true positive rate (see Figure 10) and the number of false positives (see Figure 11) is usually smaller than in the linear logistic regression model. If the number of influential variables increases, the TPR decreases even stronger than in the linear logistic model (Figure 12). However, this effect can be partially attributed to the constant *R* ^2^ value, which leads to a decreased signal per variable with increasing number of influential variables. The effect of the number of selected variables per boosting run *q* on the TPR is similar to the setting above, yet, with an earlier maximum selection frequency (Figure 13). It seems that the additive model is more sensitive on *q* as the linear logistic model. For further results consult Additional file 1 (Sec. 3). Overall, one can conclude that variable selection works well in the additive regression model and the false positive rate is always controlled.

### Case study: differential phenotype expression for ASD patients versus controls

**Figure 14:**
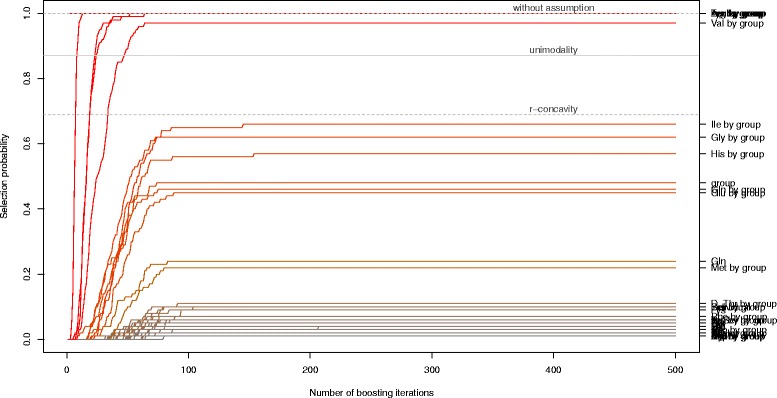
Stability selection paths. Stability selection paths, with the number of boosting iterations plotted against the relative selection frequency of the base-learners up to that iteration. One can deduce that the number of iterations was sufficiently large, as all selection paths cease to increase after approx. 150 iterations. The solid horizontal gray line is the threshold value with unimodality assumption (*π*_thr_=0.87), the dashed gray lines represent the threshold values with r-concavity assumption (*π*_thr_=0.69) and without assumption (*π*_thr_=1).

**Figure 15:**
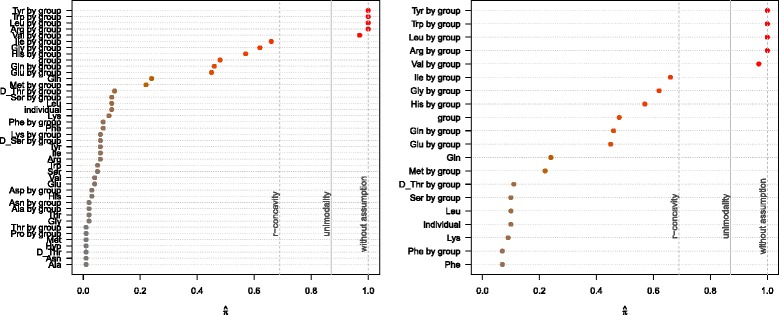
Maximum selection frequency. The maximum selection frequency $\hat {\pi }$ for all (selected) base-learners (left) and for the top 20 base-learners (right) as determined by stability selection. The solid vertical gray lines depict the threshold value with unimodality assumption (*π*_thr_=0.87), the dashed gray lines represent the threshold values with r-concavity assumption (*π*_thr_=0.69) and without assumption (*π*_thr_=1).

The stability paths resulting from the model for differential pathways in ASD patients can be found in Figure 14. The maximum inclusion frequencies for all selected base-learners and for the top scoring base-learners can be found in Figure 15. Tyrosine (Tyr), tryptophan (Trp), leucine (Leu) and arginine (Arg) all had a selection frequency of 100%. Valine (Val) was selected in 97% of the models. Without assumptions, only the amino acids with 100% selection frequency were considered to be stable. Under the unimodality assumption, valine was additionally termed stable. Together with the sharp decline in the selection frequency, we would thus focus on these first five amino acids.

The results of our analysis using stability selection confirmed the abnormal metabolism of the amino acid tryptophan in ASD cells reported by [42], who used Significance Analysis of Microarrays (SAM) [48] to assess differential expression. Additionally, the utilization of other amino acids seemed to be affected, although on a milder level. When weighted for the size of the effect, we noticed in ASD patients an overall decreased utilization of tryptophan (−0.273 units on the logarithmic scale), tyrosine (−0.135), and valine (−0.054). On the other hand, we registered an increased rate for the metabolic utilization of arginine (+0.084) and leucine (+0.081). These findings suggest an abnormal metabolism of large amino acids (tryptophan, tyrosine, leucine, and valine), which might be related to impaired transport of those molecules across the cellular membrane. Separately, a screening by Sanger sequencing was performed on the coding regions of *SLC3A2*, *SLC7A5*, and *SLC7A8*, the genes coding the subunits of the Large Amino acid Transporter (LAT) 1 and 2, in 107 ASD patients (including the ones reported in this paper; Boccuto, unpublished data; primer sequences are given as Additional file 3). Overall, potentially pathogenic mutations were detected in 17/107 ASD patients (15.9*%*): eight in *SLC3A2*, four in *SLC7A5*, and five in *SLC7A8*. We also evaluated the transcript level for these genes by expression microarray in 10 of the 17 ASD patients reported in this paper and 10 controls. The results showed that all the ASD patients had a significantly lower expression of *SLC7A5* (*p* value =0.00627) and *SLC7A8* (*p* value =0.04067). Therefore, we noticed that 27/107 ASD patients (25.2*%*) had either variants that might affect the LATs function or reduce the level of transcripts for the transporters’ subunits. When we correlated the metabolic data collected by the Phenotype Microarrays with those findings, we noticed that all of these patients showed reduced utilization of tryptophan. Additionally, eight out of the twelve patients who were screened with the whole metabolic panel showed significantly reduced tyrosine utilization in at least 25 of the 27 wells containing this amino acid, seven had a reduced utilization of valine in at least 29/34 wells, and five had a reduced metabolism of leucine in at least 27/31 wells. These data are concordant with the present findings as they suggest an overall problem with the metabolism of large amino acids, which might have important consequences in neurodevelopment and synapsis homeostasis, especially if one considers that such amino acids are precursors of important compounds, such as serotonin, melatonin, quinolinic acid, and kynurenic acid (tryptophan), or dopamine (tyrosine).

## Conclusion

Stability selection proves to work well in high-dimensional settings with (many) more predictors than observations. It adds an error control to the selection process of boosting or other high-dimensional variable selection approaches. Assumptions on the distribution of the simultaneous selection probabilities increase the number of true positive variables, while keeping the error control in most settings. As shown in our case study, complex log-linear interaction models can be used as learners in conjunction with stability selection. Additionally, more complex models such as generalized additive models or structured additive regression (STAR) models can also benefit from the combination with stability selection if model or variable selection (with a control for the number of false positives) is of major interest.

However, one should keep in mind that stability selection controls the per-family error rate, which is very conservative. Specifying the error rate such that *α*≤*P* *F* *E* *R*_max_≤*m* *α*, with significance level *α* and *m* hypothesis tests, might provide a good idea for a sensible error control in high-dimensional settings with *F* *W* *E* *R*-control (*P* *F* *E* *R*_max_=*α*) and no multiplicity adjustment (*P* *F* *E* *R*_max_=*m* *α*) as the extreme cases.

Furthermore, prediction models might not always benefit from stability selection. If the error control is tight, i.e. *P* *F* *E* *R*_max_ is small, the true positive rate is usually smaller than in a cross-validated prediction model without stability selection and the prediction accuracy suffers (see also [49]). Prediction and variable selection are two different goals.

## Availability of supporting data

The ASD data set is available as a supplement to Boccuto *et al.* [42] and as boccuto_et_al in the R package opm [44-46].

### Implementation and source code

Stability selection is implemented in the add-on package stabs [50] for the statistical program environment R [51]. One can directly use stability selection on a fitted boosting model using the function stabsel(). One only needs to additionally specify two of the parameters PFER, cutoff and q. The missing parameter is then computed such that the specified type of error bound holds (without additional assumptions (assumption = "none"), under unimodality (assumption = "unimodal") or under r-concavity (assumption = "r-concave")). It is very fast and easy to change either PFER, cutoff or the assumptions for a given stability selection object if q is kept fix, as we do not need to re-run the subsampling algorithm but simply need to adjust the threshold *π*_thr_ and the error bound *P* *F* *E* *R*_max_. This fact is exploited by a special stabsel() function, which we can re-apply to stability selection objects.

Alternative stabsel() methods exist for various other fitting approaches (e.g. Lasso). By specifying a function that returns the indices (and names) of selected variables one can easily extend this framework. In general, the function stabsel_parameters() can be used to compute the missing parameter without running stability selection itself to check if the value of the parameter computed from the other two parameters is sensible in the data situation at hand.

The component-wise, model-based boosting approach is implemented in the R add-on package mboost [26,36,52]. A comprehensive tutorial for mboost is given in [27]. The R package opm [44-46] is used to store, manage and annotate the data set. Tutorials are given as vignettes.

## Additional files

Additional file 1 explanation of complementary pairs stability selection, including the error bounds for various assumptions and an interpretation of the *expected number of selected variables with low selection probability*. Section 3 displays further results from the simulation study for Gaussian additive regression models.

Additional file 2
**R source code.** The exemplary R source code can be used to analyze the ASD data. It shows how to obtain and pre-process the data using the R package opm and how to fit the models using the R package mboost. Based on the fitted model, the the R package stabs is used to run stability selection and to depict the results. Please install the latest versions of the packages opm, mboost and stabs before use.

Additional file 3
**Primers.** The file includes the sequences of the oligonucleotide primers utilized for the Sanger sequencing of coding regions and intron/exon boundaries of the three genes encoding the protein subunits of the major tryptophan transporters: *SLC3A2*, *SLC7A5*, and *SLC7A8*. Each sequence is also comprehensive of an M13 segment (in lower cases).
