# Supplementary material for: Controlling false discoveries in high-dimensional situations: boosting with stability selection
Source: BMC Bioinformatics. 2015 May 6;16:144. doi: 10.1186/s12859-015-0575-3 (PMC4464883; doi:10.1186/s12859-015-0575-3)
Supplement: Additional file 1 — explanation of complementary pairs stability selection, including the error bounds for various assumptions and an interpretation of the expected number of selected variables with low selection probability. Section 3 displays further results from the simulation study for Gaussian additive regression models. [file 12859_2015_575_MOESM1_ESM.pdf]

Supplement to

Controlling false discoveries in high-dimensional situations: Boosting with stability selection

Benjamin Hofner[*†] Luigi Boccuto [†] Markus Göker[‡]

A.1. Definitions and discussion of common error rates

There are various definitions of error rates that are used in statistics, especially in the case of multiple testing. Let m be the number of tested hypothesis, R the number of rejected hypothesis and V the number falsely rejected hypotheses as defined above (cf. Benjamini and Hochberg 1995). In our case, m is the number of predictor variables p or more general the number of base-learners in the boosting model. Commonly used error rates include the per-comparison error rate $PCER = \mathbb{E}(V)/m$, the per-family error rate $PFER = \mathbb{E}(V)$, the family-wise error rate $FWER = \mathbb{P}(V \geq 1)$, and the false discovery rate $FDR = \mathbb{E}(\frac{V}{R})$ (Benjamini and Hochberg 1995). The per-comparison error rate is the standard error rate without adjustment for multiplicity.

For a given test situation it holds that

$$PCER \leq FWER \leq PFER\,.$$

Thus, for a fixed significance level α it holds that *PFER*-control is more conservative than *FWER*-control which is in turn more conservative than *PCER*-control (Dudoit, Shaffer, and Boldrick 2003). The *FDR*, which is often used in (very) high-dimensional settings such as gene expression studies uses another error definition by relating the number of false discoveries to the number of rejected null hypotheses. One can show that in a given test situation

$$FDR \leq FWER,$$

and thus for a fixed level α, *FWER*-control is more conservative than *FDR*-control (Dudoit *et al.* 2003). In conclusion, it holds that $FDR \leq FWER \leq PFER$. Controlling the *PFER* is a (very) conservative approach for controlling errors in multiple testing situations. Hence, a procedure that controls the *PFER* at a certain level α also controls all other error rates discussed in this section at this level. Obviously the error bound will be very conservative upper bound for both the *FWER* and *FDR*.

[*]E-mail: benjamin.hofner@fau.de

[†]Department of Medical Informatics, Biometry and Epidemiology, Friedrich-Alexander-Universität Erlangen-Nürnberg, Waldstraße 6, 91054 Erlangen, Germany

[†]Greenwood Genetic Center, 113 Gregor Mendel Circle, Greenwood, SC 29646, USA

[‡]Leibniz Institute DSMZ – German Collection of Microorganisms and Cell Cultures, Inhoffenstraße 7b, 38124 Braunschweig, Germany

The standard approach for hypotheses testing, neglecting multiplicity, would be to specify a bound for the per-comparison error rate by using a significance level α, e.g. $\alpha = 0.05$. This is equal to specifying $PFER_{\max} \leq m\alpha$. This provides some guidance on how to choose an upper bound for the *PFER*: Usually, $\alpha \leq PFER_{\max} \leq m\alpha$ seems a good choice, where $PFER_{\max} = \alpha$ would (conservatively) control the *FWER* on the level α, while $PFER_{\max} = m\alpha$ would control the unadjusted per-comparison error rate on the level α. Everything in between can be considered to control the *PCER* on the level α "with some multiplicity adjustment".

A.2. Improved version of stability selection

A modification of stability selection was introduced by Shah and Samworth (2013). One major difference to the original stability selection approach is that instead of using B independent subsamples of the data, Shah and Samworth (2013) use $2B$ complementary pairs: One draws B subsamples of size $\lfloor n/2 \rfloor$ from the data and uses, for each subsample, the remaining observations as a second complementary subsample.

More importantly, error bounds are theoretically derived that hold without assuming exchangeability of the noise variables (and without assuming that the original selection procedure is not worse than random guessing). The drawback of being able to drop the exchangability assumption and the assumption that the selection of boosting is not worse than random guessing is that the modified bounds do not control the per-family error rate, but the *expected number of selected variables with low selection probability*

$$\mathbb{E}(|\hat{\mathrm{S}}_{\text{stable}} \cap L_\theta|), \tag{1}$$

where $\hat{\mathrm{S}}_{\text{stable}}$ denotes the set of variables selected by stability selection, and $L_\theta = \{j : \hat{\pi}_j \leq \theta\}$ denotes the set of variables that have a low selection probability under $\hat{S}_{\lfloor n/2 \rfloor}$, i.e. a selection probability below θ in one boosting run on a subsample of size $\lfloor n/2 \rfloor$. Usually, this threshold for low selection probabilities is chosen as $\theta = \frac{q}{p}$, i.e. the average fraction of selected variables. Thus, this error rate represents the expected number of variables that are unlikely to be selected but are selected.

Here, the selection probability $\hat{\pi}_j$ (Eq. 5, main document) needs to be computed over all $2B$ random (complementary) subsamples. Additionally, let the simultaneous selection probability $\tilde{\pi}_j$ be defined as follows (Shah and Samworth 2013):

$$\tilde{\pi}_j := \frac{1}{B} \sum_{b=1}^{B} \mathbb{I}_{\{j \in \hat{S}_b^1\}} \cdot \mathbb{I}_{\{j \in \hat{S}_b^2\}}, \tag{2}$$

where $\mathbb{I}_{\{j \in S\}}$ is the indicator function which is one if $j \in S$ and zero otherwise. $\hat{S}_b^1$ is the set of selected variables on the bth random subset of size $\lfloor n/2 \rfloor$ and $\hat{S}_b^2$ is the selection on the complementary pair of this random subset. Note that both sets of selected variables are derived with the original learning procedure without applying the stability selection threshold so far.

Shah and Samworth (2013) derive three error bounds for the *expected number of low selection probability variables*:

(E1) A worst case error bound is derived for all $\pi_{\text{thr}} \in (0.5, 1]$:

$$\mathbb{E}(|\hat{\mathrm{S}}_{\text{stable}} \cap L_\theta|) \leq \frac{\theta}{2\pi_{\text{thr}} - 1}\, \mathbb{E}(|\hat{S}_{\lfloor n/2 \rfloor} \cap L_\theta|) \leq \frac{\theta}{2\pi_{\text{thr}} - 1}\, q$$

If $\theta = \frac{q}{p}$, this error bound is equal to the error bound of Meinshausen and Bühlmann (2010) (Eq. 6, main document) but does not require that the exchangeability and "not worse than random guessing" assumptions hold.

(E2) A second, tighter, error bound assumes that the simultaneous selection probabilities $\tilde{\pi}_j$ have a unimodal probability distribution for all $j \in L_\theta$. If additionally $\theta \leq 1/\sqrt{3} \approx 0.577$ holds, the error bound can be written as

$$\mathbb{E}(|\hat{S}_{\text{stable}} \cap L_\theta|) \leq \frac{\theta}{c(\pi_{\text{thr}}, B)} \mathbb{E}(|\hat{S}_{\lfloor n/2 \rfloor} \cap L_\theta|) \leq \frac{\theta}{c(\pi_{\text{thr}}, B)} q$$

with constant

$$c(\pi_{\text{thr}}, B) = \begin{cases} 2\left(2\pi_{\text{thr}} - 1 - \frac{1}{2B}\right) & \text{if } \pi_{\text{thr}} \in (c_{\text{min}}, \frac{3}{4}] \\ \frac{1+1/B}{4\left(1-\pi_{\text{thr}}+\frac{1}{2B}\right)} & \text{if } \pi_{\text{thr}} \in (\frac{3}{4}, 1], \end{cases}$$

and $c_{\text{min}} = \min(\frac{1}{2} + \theta^2, \frac{1}{2} + \frac{1}{2B} + \frac{3}{4}\theta^2)$. One needs to further assume that $\pi_{\text{thr}} \in \left\{\frac{1}{2} + \frac{2}{2B}, \frac{1}{2} + \frac{3}{2B}, \ldots, 1\right\}$ for the bound to hold. However, this is no restriction in practice, as for typical values of B such as $B = 50$ or $B = 100$, all values of $\pi_{\text{thr}} \geq 0.51$ in steps of 0.01 or $\pi_{\text{thr}} \geq 0.505$ in steps of 0.005, respectively, are permitted.

(E3) The third error bound assumes that the simultaneous selection probabilities $\tilde{\pi}_j$ have an r-concave probability distribution with $r = -\frac{1}{2}$ and that the selection probabilities $\hat{\pi}_j$ have an r-concave probability distribution with $r = -\frac{1}{4}$ for all $j \in L_\theta$. With f_j being the distribution of $\tilde{\pi}_j$ and g_j being the distribution of $\hat{\pi}_j$ this is equivalent to the assumptions that $f_j^{-1/2}$ and $g_j^{-1/4}$ must be convex. The r-concavity assumption lies in between unimodality and the stronger log-concavity assumption. For details on r-concavity we refer to Shah and Samworth (2013). If the r-concavity assumption holds, the error bound can be further refined as

$$\begin{aligned} \mathbb{E}(|\hat{S}_{\text{stable}} \cap L_\theta|) &\leq \min\left\{D\left(2\pi_{\text{thr}} - 1; \theta^2, B, -\frac{1}{2}\right), D\left(\pi_{\text{thr}}; \theta, 2B, -\frac{1}{4}\right)\right\} |L_\theta| \\ &\leq \min\left\{D\left(2\pi_{\text{thr}} - 1; \theta^2, B, -\frac{1}{2}\right), D\left(\pi_{\text{thr}}; \theta, 2B, -\frac{1}{4}\right)\right\} p. \end{aligned}$$

The function $D(\xi; \theta, B, r)$ denotes the maximum of the probability $P(X \leq \xi)$ with $\mathbb{E}(X) \leq \theta$ over all r-concave random variables X on a discrete support $\{0, 1/B, 2/B, \ldots, 1\}$. For details see Shah and Samworth (2013, Appendix A.4).

With these additional assumptions we get much tighter error bounds. The reason for tighter bounds can be found in the application of refined bounds in Markov's inequality that make use of the distributional assumptions. Markov's inequality is used on the simultaneous selection probabilities $\tilde{\pi}_j$ in the derivation of the error bounds (see Shah and Samworth 2013, App. A.1–A.3).

One should be aware that the assumptions are on the *distribution* of the selection probabilities and not on the selection probability itself. The unimodality assumption seems to generally hold in practice. The r-concavity assumption may fail, if the number of subsamples B increases, since as B increases, r-concavity requires an increasing number of inequalities to hold for the distribution of $\tilde{\pi}_j$. However, the same problem does not occur for the unimodal bound, and when $B = 50$, the bounds constructed using the r-concavity assumption seem to hold in a wide variety of scenarios

(Shah, 2014, personal communication; see also results of the simulation study).

A.2.1. Interpretation of $\mathbb{E}(|\,\hat{S}_{\text{stable}} \cap L_\theta|)$

If the exchangeability assumption holds and the selection procedure is not worse than random guessing, then all noise variables have a "below average" selection probability. Hence, the low selection probability variables will include all noise variables, i.e. $L_\theta = N$. Controlling the *expected number of selected variables with low selection probability* is thus in this case identical to controlling the expected number of false positives:

$$\mathbb{E}(|\,\hat{S}_{\text{stable}} \cap L_\theta|) = \mathbb{E}(|\,\hat{S}_{\text{stable}} \cap N|) = \mathbb{E}(V).$$

Stability selection can consequently be thought to control the per-family error rate in all three cases (E1) – (E3). On the other hand, if exchangeability does not hold, this means that we have "special" noise variables, e.g., noise variables that are stronger correlated with signal variables than other noise variables. If this correlation is so strong that a variable is selected with "above average selection probability", it is difficult to think of this variable as noise variables anyway. Thus controlling the *expected number of selected variables with low selection probability* is again similar or even practically identical to controlling the expected number of false positives.

B. Additional results for the simulation study with Gaussian additive models

Figure 1 displays the dependency of the true positive rate on the number of observations n. The dependency of the number of false positives on n is displayed in Figure 2, while the influence of the number of influential variables p_{infl} is depicted in Figure 3. The number of false positives dependent on q is given in Figure 4.

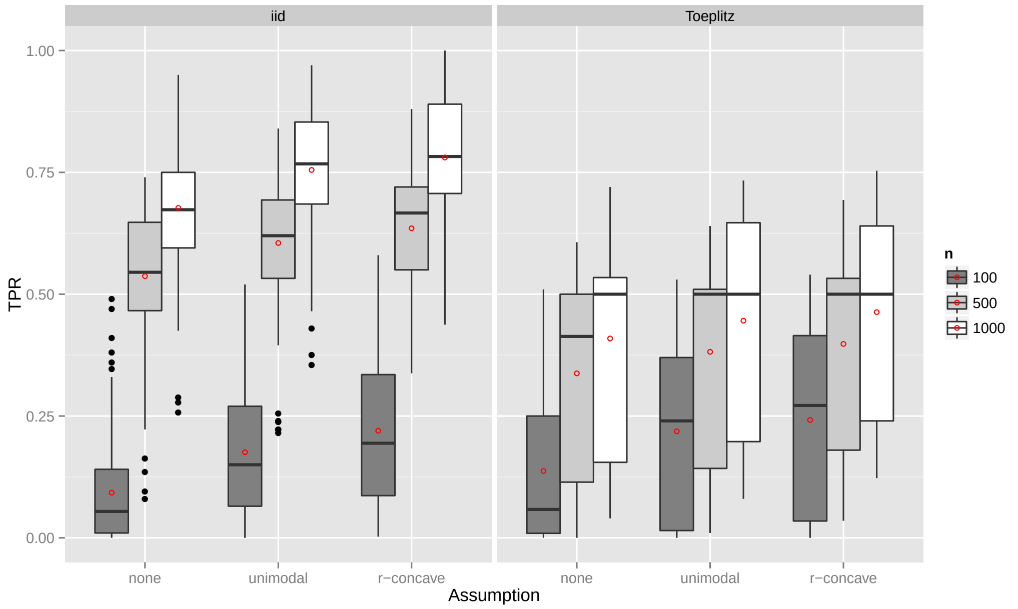

Figure 1: True positives rates by the number of observations n – Gaussian additive regression model. Boxplots for the true positives rates (TPR) for all simulation settings with separate boxplots for different numbers of observations (n), the correlation settings (independent predictor variables or Toeplitz design), and the assumptions used to compute the error bound. Each observation in the boxplot is the average of the 50 simulation replicates. The open red circles represent the average true positive rates.

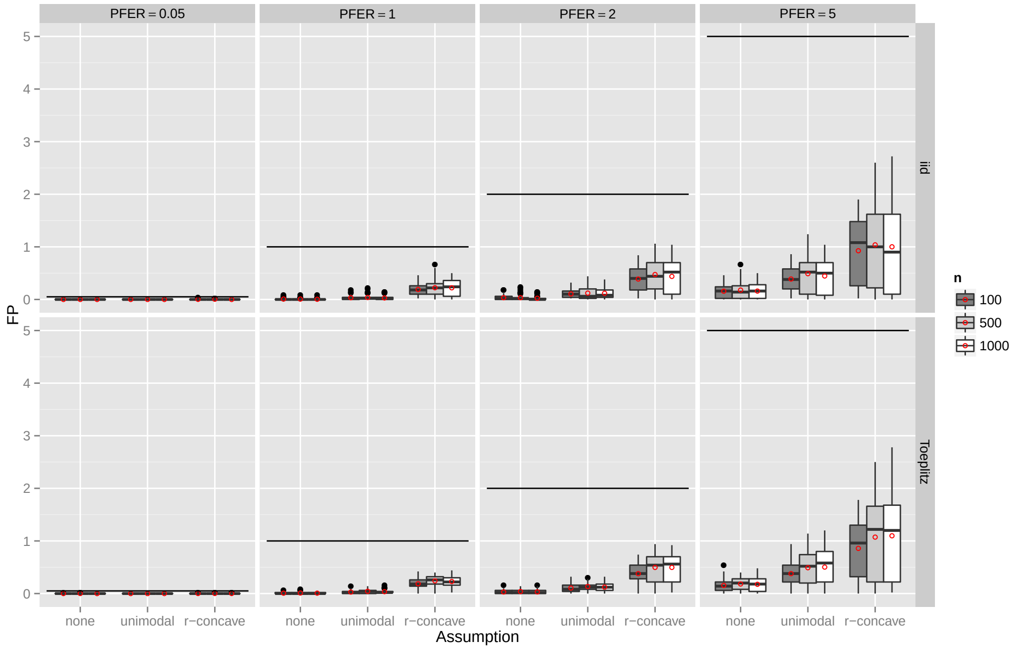

Figure 2: Number of false positives by the number of observations n – Gaussian additive regression model. Boxplots for the number of false positives (FP) for all simulation settings with separate boxplots for different numbers of observations (n), the correlation settings (independent predictor variables or Toeplitz design), the *PFER*, and the assumptions used to compute the error bound. Each observation in the boxplot is the average of the 50 simulation replicates. The open red circles represent the average number of false positives.

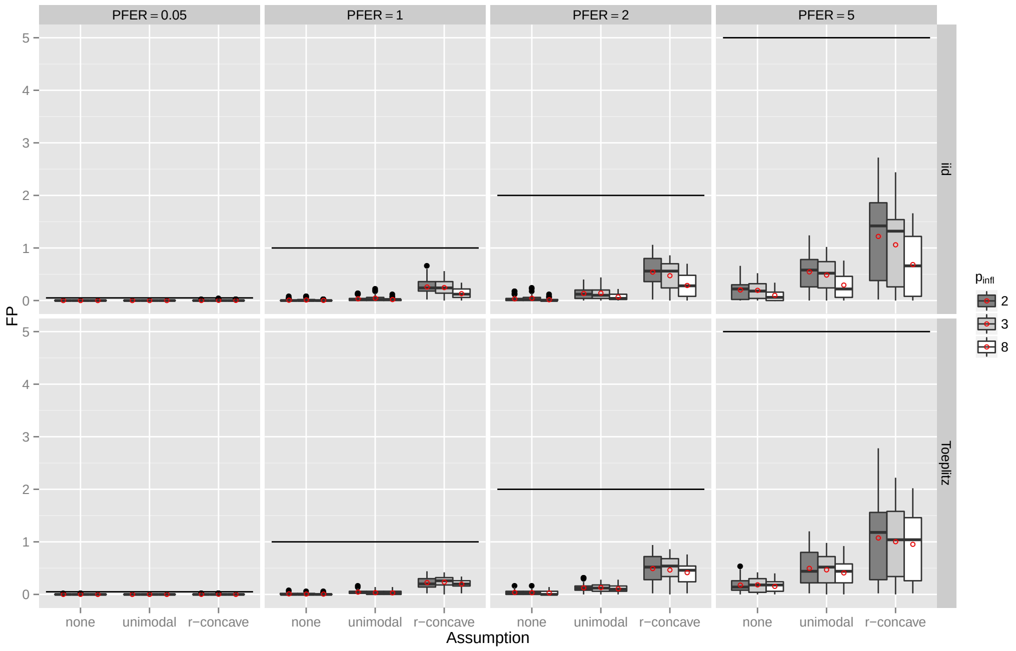

Figure 3: Number of false positives by the number of influential variables p_{infl} – Gaussian additive regression model. Boxplots for the number of false positives (FP) for all simulation settings with separate boxplots for different numbers of influential variables (p_{infl}), the correlation settings (independent predictor variables or Toeplitz design), the *PFER*, and the assumptions used to compute the error bound. Each observation in the boxplot is the average of the 50 simulation replicates. The open red circles represent the average number of false positives.

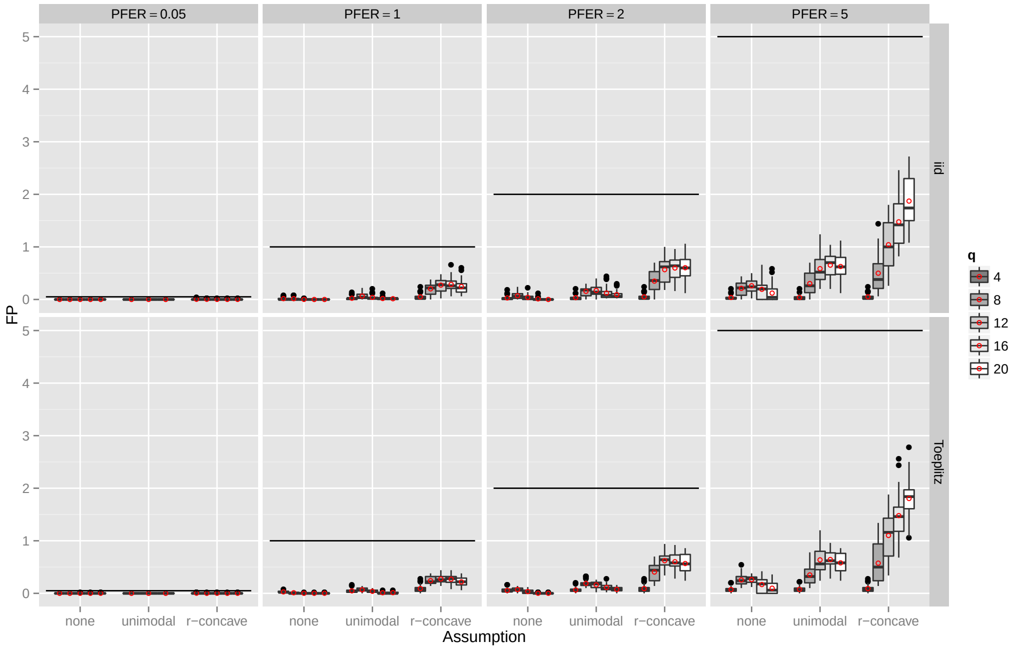

Figure 4: Number of false positives by the number of selected variables per boosting run q – Gaussian additive regression model. Boxplots for the number of false positives (FP) for all simulation settings with separate boxplots for different numbers of selected variables per boosting run (q), the correlation settings (independent predictor variables or Toeplitz design), the *PFER*, and the assumptions used to compute the error bound. Each observation in the boxplot is the average of the 50 simulation replicates. The open red circles represent the average number of false positives.

References

Benjamini Y, Hochberg Y (1995). "Controlling the false discovery rate: A practical and powerful approach to multiple testing." *Journal of the Royal Statistical Society. Series B (Methodological)*, **57**, 289–300.

Dudoit S, Shaffer JP, Boldrick JC (2003). "Multiple hypothesis testing in microarray experiments." *Statistical Science*, **18**, 71–103.

Meinshausen N, Bühlmann P (2010). "Stability selection (with discussion)." *Journal of the Royal Statistical Society: Series B (Statistical Methodology)*, **72**, 417–473.

Shah RD, Samworth RJ (2013). "Variable selection with error control: another look at stability selection." *Journal of the Royal Statistical Society: Series B (Statistical Methodology)*, **75**, 55–80.
